# Re-emergence of mayaro virus and coinfection with chikungunya during an outbreak in the state of Tocantins/Brazil

**DOI:** 10.1186/s13104-022-06153-6

**Published:** 2022-08-03

**Authors:** Robson dos Santos Souza Marinho, Rodrigo Lopes Sanz Duro, Débora Bellini Caldeira, Juliana Galinskas, Mânlio Tasso Oliveira Mota, James Hunter, Maria da Aparecida Rodrigues Teles, Flávio Augusto de Pádua Milagres, Ricardo Sobhie Diaz, Fernando Shinji Kawakubo, Shirley Vasconcelos Komninakis

**Affiliations:** 1 Retrovirology Laboratory, Federal University of São Paulo, São Paulo City, São Paulo 04039-032 Brazil; 2 Central Public Health Laboratory of Tocantins (LACEN/TO), Palmas City, Tocantins 77016-330 Brazil; 3 Institute of Biological Sciences, Federal University of Tocantins, Palmas City, Tocantins 77001-090 Brazil; 4 Tocantins Health Department, Palmas City, Tocantins 77453-000 Brazil; 5 Faculty of Philosophy, Letters and Human Sciences, University of São Paulo, São Paulo City, São Paulo 05508-000 Brazil; 6 Faculty of Medicine (FMUSP), Institute of Tropical Medicine, University of São Paulo, São Paulo City, São Paulo 05403-000 Brazil

**Keywords:** Arbovirus, Molecular screening, Coinfection, Mayaro, Chikungunya, Brazil

## Abstract

**Objective:**

To perform a molecular screening to detect infections by the mayaro virus and possible coinfections with Chikungunya during an outbreak in the state of Tocantins/Brazil in 2017.

**Results:**

Of a total 102 samples analyzed in this study, 6 cases were identified with simultaneous infection between mayaro and chikungunya viruses (5.88%). In these 6 samples, the mean Cycle threshold (Ct) for CHIKV was 26.87 (SD ± 10.54) and for MAYV was 29.58 (SD ± 6.34). The mayaro sequences generated showed 95–100% identity to other Brazilian sequences of this virus and with other MAYV isolates obtained from human and arthropods in different regions of the world. The remaining samples were detected with CHIKV monoinfection (41 cases), DENV monoinfection (50 cases) and coinfection between CHIKV/DENV (5 cases). We did not detect MAYV monoinfections.

## Introduction

The arbovirus (*arthropod–borne viruses*) emergence and re-emergence has become increasingly frequent in countries of the tropical and subtropical regions of the world [1, 2]. Cases of coinfections involving different arboviruses during outbreaks are becoming common in areas where these viruses are co-circulating [3–5]. The arboviruses are the main causative agents of infectious diseases of public health importance [6]. The environmental conditions, vector density and migration and immigration processes contribute to the spread and maintenance of these viruses in nature [7].

The chikungunya (CHIKV) and mayaro (MAYV) viruses are endemic arboviruses in Brazil, belongs to the family *Togaviridae* (genus *Alphavirus)*. These viruses cause an acute febrile illness nonspecific that can lead to severe and debilitating clinical conditions [8]. Currently, three genotypes different for each of these arbovirus have been reported, being them: West Africa, East-Central South Africa (ECSA) and Asian to CHIKV [9] and the genotypes D (widely dispersed), L (limited) and N (new) to MAYV [10].

The CHIKV was identified for the first time in Brazil in 2014 [11]. The Asian and ECSA genotypes entered in country through the northern (Amapá state) and northeast (Bahia state) regions, respectively [12]. MAYV was reported initially in the Amazon region and later its circulation was notified in other areas of Brazil, including the states of Goiás, Mato Grosso, São Paulo, and Rio de Janeiro [13–16]. Currently, both viruses circulate in country causing outbreaks or sporadic cases of infections in humans [17, 18].

Some studies have shown cases of coinfection between MAYV and other arboviruses. One recent paper by Aguilar-Luis [19] sought to show the emergence of the MAYV and cases of coinfection with Dengue virus (DENV) in Peru. The authors demonstrated that of a total of 496 samples analyzed, the prevalence of people coinfected with DENV/MAYV was 6.4%. In a systematic review showing the frequency and clinical presentation of coinfections involving the Zika virus, a study reported a single case of coinfection between this virus and MAYV [20]. However, there are scarce studies reporting coinfection between MAYV and CHIKV.

In Brazil, the co-circulation of these arboviruses has been demonstrated, but coinfections between MAYV/CHIKV are still little documented. This reinforces the need for differential diagnosis for MAYV during CHIKV outbreaks. Thus, our study had the objective of to perform a molecular screening to detect infections by the mayaro virus and possible coinfections with chikungunya during an outbreak occurred in the state of Tocantins/Brazil in 2017.

## Main text

### Methods

#### Study design

In this cross-sectional observational study, we analyzed 102 serum samples obtained from patients who consulted different health units in the state of Tocantins with symptoms of arboviral infection. Initially, we performed a screening for the detection of zika, dengue and chikungunya viruses [21]. Here, we report a screening performed for the detection of MAYV. Clinical and demographic information of the patients were described by the health units and sent to the LACEN de Palmas/Tocantins. Afterwards, these data were sent to the Retrovirology Laboratory. The samples analyzed were collected between the months of January to August of 2017.

#### Study area

**Fig. 1:**
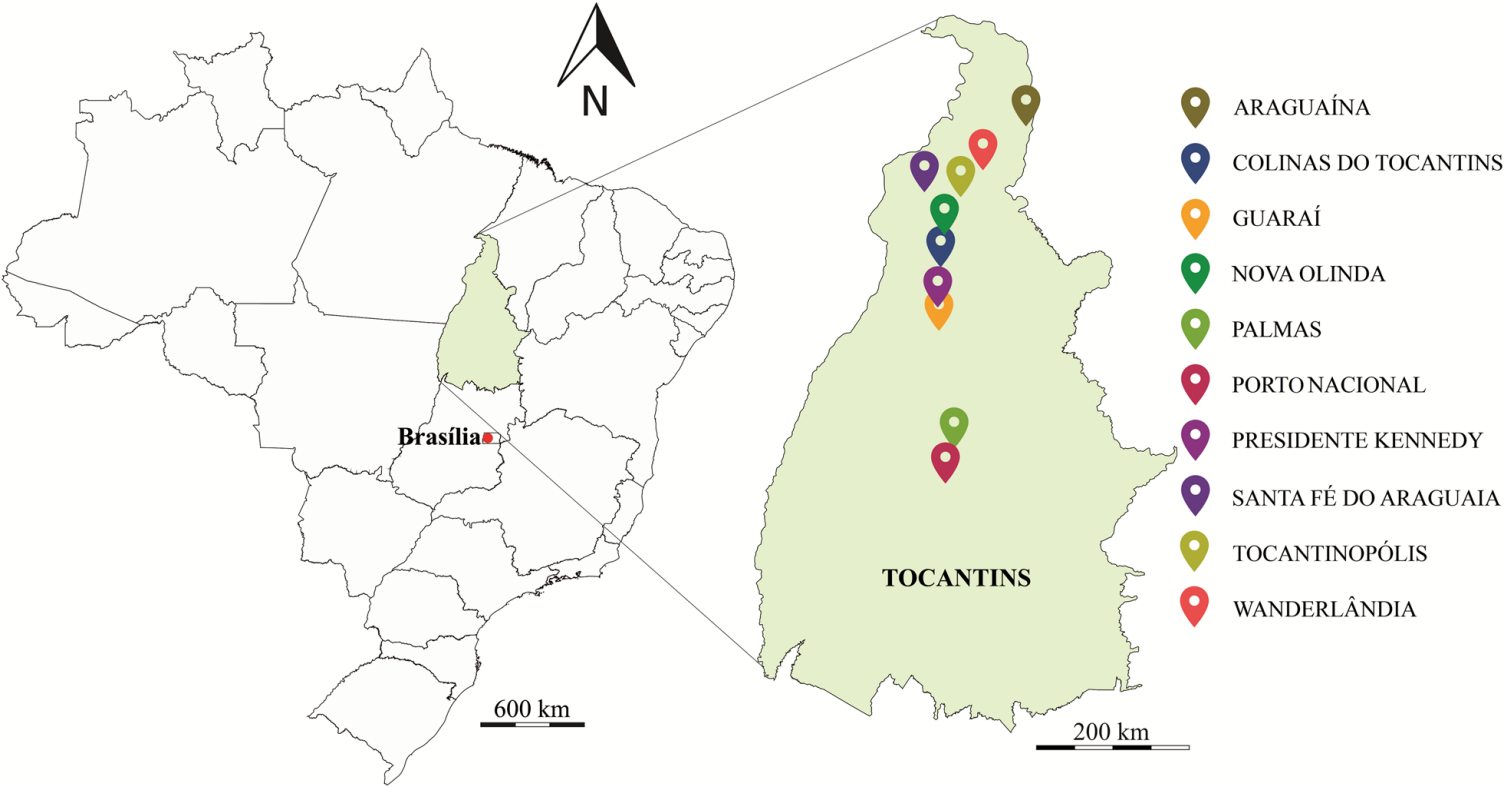
Location of the study area (state of Tocantins/TO) and of the municipalities where the samples were collected (collection points). This map was built using the program QGIS, v. 2.18

Tocantins is a Brazilian state located in the northern region of the country. With an area of 277,720.520 km^2^, it borders the states of Goiás (South), Piaui (East), Maranhão (Northeast), Bahia (Southeast), Pará (Northwest) and Mato Grosso (Southwest) (Fig. 1) [22].

#### Ethics statement

This study was approved by the Research Ethics Committee of the Federal University of Sao Paulo [21] (CAAE: 18908719.2.0000.5505). All patients enrolled signed an informed written consent. The patients’ personal information was anonymized before the data was accessed. This study accessed the information of the patients on demographic characteristics, clinical signs, and symptoms***.***

#### Inclusion criteria

The study participants were individuals with more than 18 years of age, included both sexes and presented compatible symptoms of arboviral infections (fever, arthralgia, exanthema, headache, myalgia, nausea, retro-orbital pain, generalized body pain, among other clinical aspects and were tested within 8 days of the onset of symptoms, following criteria established by the World Health Organization [23] and Centers for Disease Control and Prevention [24].

#### Detection molecular of dengue, chikungunya and mayaro

All 102 samples were analyzed by molecular diagnosis. First, the RNA viral was obtained from 200 μL of serum.

samples using the QIAamp Viral RNA Mini Kit, following the manufacturer’s recommendations. Previously, we had performed RT-qPCR assay for detection of dengue and chikungunya using the GoTaq^®^Probe1-Step RT-qPCR System following recommendations of the manufacturer. The primers and probes were previously described in Alm et al. [25] and Cecilia et al. [26] for dengue and chikungunya, respectively. For the detection of MAYV, complementary DNA (cDNA) was generated using GoScript™ Reverse Transcriptase, following the manufacturer’s recommendations. Immediately, these cDNAs were used in the Real Time RT-PCR assay with SYBR^™^ Green PCR Master Mix using a pair of primers described by Mourão et al. [27] for identification of this pathogen. Strains of DENV, CHIKV and MAYV obtained from cell culture and confirmed by sequencing were used as positive controls. Ultra-pure water served as a negative control and Ribonuclease P was used as internal control. All reactions were performed in duplicate. The reactions were carried out in the ABI 7500 Real Time PCR system. In this study, serological tests were not performed.

#### Confirmation of MAYV by sequencing

All the positive samples for MAYV were sequenced using Sanger-based sequencing. Firstly, conventional PCR was performed using protocol, including primers and thermal profiles previously established [27], generating a product of 201 base pairs (E1 glycoprotein). The cDNA previously generated was used as target. All reactions included positive and negative controls to ensure the reliability of the reaction. All the amplified products were sequenced using BigDye^™^ Terminator v3.1 Cycle Sequencing Kit. The sequences were edited and aligned using Sequencher v.5.0.1 and BioEdit v.7.1. All sequences generated were subjected to the BLAST (https://blast.ncbi.nlm.nih.gov/Blast.cgi) analysis using the megablast algorithm for highly similar sequences [28] and deposited in GenBank.

#### Statistical analysis

The percentage of women and men were determined. The mean age and Cycling threshold (Ct) with the respective standard deviations (SD) were calculated. The prevalence was presented as absolute values with 95% confidence intervals (95% CI). All analyses were realized in the IBM SPSS software version 21.0.

### Results

Of the total number of samples analyzed in this study, we detected 6 cases of MAYV, and all these cases presented coinfection with CHIKV. The clinical features of these patients are shown in Table 1. MAYV monoinfections were not detected. Of the remaining samples, 41 cases of CHIKV monoinfection were detected, 50 cases of DENV monoinfection and 5 coinfections between CHIKV/DENV. The clinical and demographic characteristics of these participants are shown in Table 1. These findings were described in one study previously published by our group [21].

**Table 1:** Clinical and demographic characteristics of the CHIKV or DENV monoinfected patients and CHIKV/DENV coinfected

Clinical and demographic characteristics	Patients (%)*		
	CHIKV (n = 41)	DENV (n = 50)	CHIKV/DENV (n = 5)
Sex			
Male	18 (43.9)	16 (32.0)	2 (40.0)
Female	23 (56.1)	34 (68.0)	3 (60.0)
Age (years)			
18–24	9 (21.9)	12 (24.0)	1 (20.0)
25–31	12 (29.2)	9 (18.0)	–
32–38	16 (39.0)	21 (42.0)	3 (60.0)
39–45	4 (9.7)	8 (16.0)	1 (20.0)
Location of residence			
Araguaína	8 (19.5)	6 (12.0)	3 (60.0)
Colinas do Tocantins	–	7 (14.0)	–
Guaraí	–	6 (12.0)	–
Nova Olinda	–	2 (4.0)	–
Palmas	14 (34.1)	10 (20.0)	2 (40.0)
Porto Nacional	5 (12.2)	7 (14.0)	–
Presedente Kennedy	2 (4.9)	1 (2.0)	–
Santa fé do Araguaína	1 (2.4)	1 (2.0)	–
Tocantinópolis	–	2 (4.0)	–
Wanderlândia	11 (26.8)	8 (16.0)	–
Signs and symptoms			
Fever	38 (92.6)	41 (82.0)	5 (100)
Arthralgia	39 (95.1)	34 (82.9)	5 (100)
Exanthema	36 (87.8)	42 (84.0)	5 (100)
Headaches	37 (90.2)	39 (78.0)	5 (100)
Myalgia	34 (82.9)	43 (86.0)	3 (60.0)
Diarrhea	6 (14.6)	6 (12.0)	–
Non-purulent conjunctivitis	14 (34.1)	4 (8.0)	5 (100)
Retro-orbital pain	11 (26.8)	50 (100)	5 (100)
Abdominal pain	5 (12.1)	36 (72.0)	3 (60.0)
Edema	4 (9.7)	–	–
Hemorrhagic manifestations	–	32 (64.0)	–
Generalized body pain	38 (92.6)	49 (98.0)	–
Nausea	16 (39.0)	32 (64.0)	5 (100)
Vomiting	14 (34.1)	25 (50.0)	3 (60.0)
Leukopenia	11 (26.8)	16 (32.0)	5 (100)

^*^Number of patients (% of clinical and demographic characteristics)

We observed a prevalence of 5.88% (95% CI 1.78–8.96) patients confirmed with simultaneous infection between CHIKV and MAYV. The mean age of these patients was 34.16 years (SD = 21.13). Of these six patients, 2 (33.33%) were women and 4 (66.67%) were men. The mean Cycle threshold (Ct) for CHIKV was 26.87 (SD = 10.54) and for MAYV was 29.58 (SD = 6.34). These individuals were residents of the municipalities of Colinas do Tocantins, Wanderlândia and Palmas, as described in Table 2.

**Table 2:** Clinical aspects of the six patients detected with simultaneous infection between CHIKV and MAYV in Tocantins/Brazil

Patients^a^	Sex	City	Clinical features	Genbank accessions
0190	F	Colina do Tocantins	Fever, headache, myalgia, arthralgia, nausea, rash	OM718766
0237	M	Wanderlândia	Fever, headache, myalgia, generalized body pain, arthralgia, nausea, rash	OM718767
0802	M	Palmas	Fever, headache, myalgia, polyarthralgia, nausea, rash, generalized body pain	OM718768
0740	M	Palmas	Fever, headache, myalgia, generalized body pain, arthralgia, nausea, rash	OM718769
0264	F	Colina do Tocantins	Arthralgia, fever, headache, abdominal pain, nausea, generalized body pain	OM718770
0755	M	Palmas	Fever, headaches, myalgia, arthralgia, vomiting, generalized body pain	OM718771

^a^Patient identification numbers

The MAYV sequences generated in this study showed 95–100% identity with other; Brazilian sequences of this virus, such as HQ664947 isolated in Manaus/AM (2012), KM400591 isolated in the state of Acre (2014) and KY618130 in the state of Pará (2017). Other MAYV isolates obtained from human and arthropods in different regions of the world also showed high similarity with our sequences, as well as MK573240 from Trinidad and Tobago (1957), MK070491 from Peru (1997), MK573246 from Bolivia (1955), KJ742385 from French Guiana (2014) and KP842810 from Venezuela (2015). The six MAYV sequences obtained were deposited in GenBank and accession numbers are showed in Table 2.

### Discussion

In this study, we performed one molecular screening to detection of infections by the MAYV and identified six patients coinfected with CHIKV/MAYV in the state of Tocantins. There is still little information available on the emergence of the MAYV and cases of coinfection involving this arbovirus in Brazil, especially in the northern region of the country. This is the first report of detection of coinfection between chikungunya e mayaro viruses in State of Tocantins. Thus, our study had the objective of contribute to this field of knowledge helping to inform public policies that ensure improvements in the diagnosis of arboviruses.

The emergence of MAYV has been documented in many countries of the Latin America and Caribbean [29]. During an outbreak of febrile illness with arthralgic manifestations in Venezuela in 2010, the MAYV was identified and characterized as the causative agent of the outbreak [30]. In Haiti, between 2014 and 2015, a total of 177 blood samples obtained from children with acute febrile illness were analyzed. This screening detected a child infected with MAYV [31]. One cross-sectional study conducted with 359 serum samples from patients with suspected febrile illness in Peru, in 2017, were detected 40 participants infected with MAYV [32].

In Brazil, the circulation and sporadic cases of infection by MAYV have been reported in different regions of the country. Recently, Saatkamp et al. [33] analyzed 49 serum samples from patients in the state of Pará and detected four positive cases for MAYV. Still in the northern region of the country, another case of MAYV infection in humans was detected in the state of Acre [34]. Silveira–Lacerda et al. [35] in the city of Goiânia/Goiás, Midwest region, showed a molecular epidemiology investigation of the MAYV in febrile patients from 2017 to 2018 and of a total of 375 individuals analyzed, 26 were positive for this virus. A serological survey realized to track MAYV infections in blood donors from São Carlos in the state of São Paulo, has left evidence of the circulation of this virus also in the southeast region [36]. This shows that MAYV has a wide circulation in Brazil, and this reinforces the need of the differential diagnosis for the arboviruses of importance to public health.

An important finding in our work was the detection of the coinfection between CHIKV/MAYV. Some studies show that dual infection involving these two arboviruses can cause a severe and potentially incapacitating joint disease [37]. However, we did not observe severe symptoms in the six patients with the coinfection. Fever, headache, myalgia, arthralgia were the clinical aspects common in these people, corroborating a study which detected and phylogenetically characterized dual arbovirus infections among humans during a chikungunya fever outbreak in Haiti. In this work, the authors identified one coinfection with CHIKV/MAYV and pointed that these clinical features are common in both types of infection [38]. This shows that during an outbreak of CHIKV, only clinical criteria are not enough to differentiate infections between this virus and the MAYV, which makes necessary to realization of an effective differential diagnosis for these two arboviruses.

In conclusion, the molecular screening performed in this study was effective in detection of infections by mayaro virus as well as in the detection of coinfection with chikungunya virus during an outbreak occurred in State of Tocantins in 2017. In this way, our findings reinforce that MAYV circulates in urban areas causing infections in humans and that both viruses (MAYV and CHIKV) cause similar clinical conditions in the patients.

This shows also that clinical and epidemiological aspects alone are not enough to differentiate infections by these arboviruses, which makes necessary a differential diagnosis for MAYV during CHIKV outbreaks.

### Limitations

This combination of concomitant circulating of arbovirus in Brazil presents a major challenge in national public Health operations regarding case confirmation. The patients of this study are adults living in different cities of the state of Tocantins. However, there are some limitations in our work due the low number of clinical samples that we received. As it was a retrospective study, we could not get complete medical information.

## Data Availability

The datasets used and analyzed during the current study are available from the corresponding author on request.

## Abbreviations

95% CI: 95% Confidence Intervals
BLAST: Basic local alignment search tool
CAAE: Certificate of presentation of ethical appreciation
CDC: Centers for Disease Control and Prevention
cDNA: Complementary DNA
CHIKV: Chikungunya virus
Ct: Cycling threshold
DENV: Dengue virus
ECSA: East-Central South Africa
HDI: Human development index
IBGE: Brazilian Institute of Geography and Statistics
LACEN: Central Public Health Laboratory
MAYV: Mayaro virus
PCR: Polymerase chain reaction
SD: Standard deviation
SPSS: Statistical package for the social sciences
TO: Tocantins
WHO: World Health Organization
